# Scalds by Steam—An Account of a Railroad Accident

**Published:** 1855-01

**Authors:** 


### SCALDS BY STEAM—AN ACCOUNT OF A RAIL ROAD ACCIDENT.

It was our unfortunate lot to be a part of the same train of passengers in the cars upon the Chicago and Rock Island Railroad at the time of the sad accident which occurred at 2 o'clock on the morning of the 2d Nov., ultimo.

The character of the accident was more appalling than any other perhaps which has ever taken place upon any Railroad in the country, not so much because of the number injured as the shocking nature of the injuries. The fracture of bones, or wounds incised, contused, or lacerated, do not seem so horrible or distressing as those of scalds or burns, and especially where the hot, scalding steam is the agent in their production. No wound can present such hideous deformities or distortions than those produced in this way, where the features are seared and denuded of their skin and cellular tissue beneath, and the lips, eye lashes, and eye brows are removed and the eyes themselves scalded in their sockets and closed.

Sixty or more human beings of all ages and sexes were in the car which, when the engine was thrown from the rail track, was laid over upon it in a position so as to receive the whole amount of hot steam which had accumulated in the boiler, diffusing itself through and among the unfortunate inmates. Two children were taken from the car dead, while the number of twenty, or more, were so severely injured by the inhalation of the scalding steam and its effects upon large portions of the surface of the body, as to place the sufferers in the most excruciating agony and in a most hopeless condition.

The surfaces denuded of their skin covering, often including much of the cellular tissue beneath, which hung in masses and in shreds from their faces, their hands, the points of their fingers and their arms, were left of a deep red color, as if the blood was in a condition of stasis in the capillary vessels and having lost its watery constituent. So large were the abraded surfaces and so extensive the destruction of the capillary circulation, that the blood was forced upon the internal and vital organs. From its contiguity to the heart and because of the great shock upon the nervous system and the expenditure of the vital forces, the brain suffered particularly. Then again there was superadded to these the imperfect æration of the blood resulting from the lesion of the capillary bronchia, the consequent congestion upon the lungs themselves favoring the production of these symptoms of such exaggerated import as not to be easily overcome, so that in a few days several of them sunk down and died. Such was the lesion of the bronchia of the lungs in one case that the vesicular murmur was not detected in any region of the anterior surface of the throax, and it was very difficult to arouse him from the deep coma into which he had fallen. Toward the close there was cyanosis of the face and the upper and lower extremities, and yet notwithstanding the great extent of these multiplied injuries his staminal powers resisted for three days, when the struggle ceased and death triumphed. With some modifications in the symptoms the others, who died within the same period, presented similar phenomena.—With several of them there was liberal effusion in the cellular tissue, particularly the face and extremities. The tongue in a few of them we observed to be swollen, red, in some instances dry, and in one case, which we could examine, the whole buccal cavity, with the fauces, was highly vascular, and in the lattercovered with ash colored spots, as if the mucous lining was about to slough. There was in the worst cases, and those particularly who had suffered by the inhalation of the scalding steam, a secretion of muco purulent matter streaked and rusty with blood globules, which was of difficult and painful expectoration. We did not observe any cough, which we accounted for because of the loss of sensibility from great lesion or destruction of the mucous lining of the bronchia.

In the worst cases where there were extensive abrasions of the surface, one of the prognostic symptoms or means was denied us. The hands and arms being deeply abraded, the artery at the wrist could not be examined, and the only knowledge to be gleaned of the condition of the circulation was by the radial or ulnar, just below the division of the brachial, if the foot were unhurt, the pedal continuation of the anterior tibial artery, or otherwise with the ear over the region of the heart. No reaction to any appreciable or favorable extent took place with those who died within the time above alluded to, as the abraded surfaces continued of a pale and cadaveric appearance.

The treatment consisted of external dressings to the abraded surfaces, of those showing reaction to have taken place, of a soothing character; while to those indisposed to react, they were of a more stimulating nature. The ol. terebinth warmed was applied, or it was combined with ol. olive, or the liniment composed of lime-water and linseed oil. In all cases where there were blebs of serum, they were punctured at their base, the serum evacuated and the cuticle allowed to fall down and remain to cover and protect the part from the mischievous action of the atmospheric air. In some cases where the parts had so far lost their vitality as to demand the application of a direct stimulant, the skin was removed extensively for that purpose. In some instances large portions, including deeper tissue, were removed.

To quiet the nervous system anodynes were used, and to sustain the flagging and failing vital powers, stimulants, with nourishing food, was prescribed and given. As an organic and diffusible stimulent, ol. turpentine in doses of 15 drops in the form of emulsion, was exhibited every two, three, and four hours. This was very desirable also, because of its well known power of imparting tonicity to the blood vessels, which were wanting in this particular. The circulation in the vital organs was in those cases struggling in its round in consequence of the enfeebled state of the vessels, or the want of normal tonicity, from the great loss of the biotic and dynamic forces, which condition would favor congestion and accumulations. The condition of mucous tissue of the bronchia demanded its use, and those suffering from effusion would be benefited by its diuretic powers.

These cases, in many of their features, resembled adynamic erysipelas, with the exception that there was previously no blood poisoning, although it became so subsequently in consequence of the imperfect æration of the blood from the extensive lesion of the capillary bronchia. The carbon was retained in this way in the blood, still further embarrassing the circulation, and in these cases also, where a large surface was destroyed, the depuration by the sudoraparous folicles of the skin was largely diminished.

It will be seen at a glance by an intelligent Pathologist that no therapea, however timely and faithfully exhibited, would have been of permanent avail in these exaggerated cases. The great shock upon the nervous system would *per se* have had there been no other cause or causes to perpetuate the evils, sufficiently grave, but where there were superadded other sources as above referred to of portending destruction attending and following, there was no power which science could bring to bear to avert the deadly consequences.

It is but just that we should say in conclusion, that the medical corps of Joliet, who deservedly rank high in their profession, exerted themselves in behalf the sufferers who were strangers among them, and deserve great credit for their assiduity, faithfulness and the exercise of their skill to save all that was possible, and if not, to relieve anguish and suffering as far as was possible. Being thrown among them as a stranger, we were kindly and respectfully treated by them to an extent which made our stay, so far as our intercourse with them was concerned, very pleasant and agreeable ; and when we left, it was with feelings of gratitude to them, as well as to others, who did all in their power to make our detention as pleasant as under the painful circumstances it could be.

So frequent are accidents upon rail-roads at the present day, that it will become necessary, if no preventative means can be adopted to protect the travelling public from accidents and death, to establish Hospitals upon the different routes, carry a medical chest on each train, and appoint a travelling surgeon to accompany each.

